# Prognostic Significance of the Preoperative Albumin/Fibrinogen Ratio in Patients with Esophageal Squamous Cell Carcinoma after Surgical Resection

**DOI:** 10.7150/jca.58022

**Published:** 2021-06-16

**Authors:** Hongdian Zhang, Peng Ren, Mingquan Ma, Xiaolei Zhu, Kai Zhu, Wanyi Xiao, Lei Gong, Peng Tang, Zhentao Yu

**Affiliations:** 1 Department of Esophageal Cancer, Tianjin Medical University Cancer Institute and Hospital, Key Laboratory of Cancer Prevention and Therapy of Tianjin, Tianjin's Clinical Research Center for Cancer, National Clinical Research Center of Cancer, Tianjin 300060, China.; 2 Department of Thoracic Surgery, National Cancer Center, National Clinical Research Center for Cancer, Cancer Hospital & Shenzhen Hospital, Chinese Academy of Medical Sciences and PeKing Union Medical College, Shenzhen 518116, China.

**Keywords:** Esophageal squamous cell carcinoma, Albumin/fibrinogen ratio, Albumin/globulin ratio, Prognosis, Nomogram

## Abstract

**Purpose:** The purpose of the present study was to investigate the prognostic value of inflammatory and nutritional-based scores, including the albumin/fibrinogen ratio (AFR) and albumin/globulin ratio (AGR), in patients with esophageal squamous cell carcinoma (ESCC).

**Methods:** The medical records of 641 patients with resectable ESCC from our institution were retrospectively analyzed. The preoperative AFR and AGR were investigated based on serum albumin, globulin and plasma fibrinogen levels. X-tile software, Kaplan-Meier survival curves and Cox proportional hazard models were performed to identify their prognostic value. The predictive accuracy was evaluated by the concordance index (C-index), calibration plots, and decision curve analysis (DCA).

**Results:** The optimal cutoff values were 15.3 and 1.8 for AFR and AGR, respectively. Univariate survival analysis identified age, smoking history, tumor size, pT status, pN status, NLR, PLR, fibrinogen, albumin, AFR, and AGR as factors associated with overall survival. Multivariate analysis indicated that preoperative AFR (HR: 0.690, 95% CI = 0.495~0.960, *P* = 0.028), rather than other inflammation- and nutrition-based scores, was an independent predictor of overall survival. The C-index of the predicted nomogram containing AFR (C-index = 0.677) was higher than that of the nomogram without AFR (C-index = 0.656). The calibration curves showed that the predictive abilities were consistent with the actual observation results. Moreover, compared with the traditional staging system, the results of DCA showed that the nomogram had superior predictive ability and higher clinical utility.

**Conclusion:** Our preliminary study suggested that a low preoperative AFR might be a novel and valuable predictor of poor prognosis in patients with ESCC, which may be helpful for prognosis assessment, patient counseling, and therapeutic modality selection.

## Introduction

Esophageal cancer is one of the most common and aggressive gastrointestinal malignant tumors and its incidence has increased in recent years. In China, squamous cell carcinoma is the most common pathological type of esophageal cancer. Surgery-based comprehensive therapy is the best therapeutic modality for resectable esophageal squamous cell carcinoma (ESCC) 1. Despite improvements in both detection and treatment of ESCC in the past few decades, the long-term survival of patients with ESCC is disappointing, which may be due to inaccurate postoperative staging and a subsequent inappropriate choice of adjuvant treatment 2. Thus, it is important to identify new prognostic markers for the better evaluation of patients and optimization of therapy.

Several studies have demonstrated that the preoperative systemic inflammatory response and malnutrition status play critical roles in tumor progression, angiogenesis promotion, metastasis and worse prognosis in various types of malignancies, including ESCC 3, 4. Albumin is a common indicator in the evaluation of nutritional status. In addition, low serum albumin level is also a marker of the systemic inflammatory response, which can promote tumor proliferation, invasion, and migration. It has also been reported that preoperative albumin and globulin levels are potential prognostic biomarkers in various types of cancers, including non-small cell lung cancer (NSCLC), oral cancer, esophageal cancer, gastric cancer, colon cancer and gallbladder cancer 5, 6. Fibrinogen, a glycoprotein synthesized by hepatocytes, is not only an essential component of the coagulation cascade but also an acute-phase reactant reflecting the systemic inflammatory state. Studies have indicated that elevated plasma fibrinogen levels might lead to malignant tumor progression and poor prognosis in patients with gastric cancer, liver cancer, NSCLC, and gallbladder cancer 7, 8.

However, inflammation-related indicators are easily affected by other factors, and the predictive accuracy of these markers is usually low. Additionally, the best indicator that can be used for developing treatment strategies making remains unknown. Therefore, the combined detection of these inflammation-related factors may improve its clinical value in evaluating tumor prognosis. Recently, studies have reported that new parameters of inflammation, the peripheral blood albumin to globulin ratio (AGR) and albumin to fibrinogen ratio (AFR), can not only reflect the patient's nutrition, blood coagulation, and inflammation status simultaneously but also have a stronger power to improve the prognostic value of patients with lung cancer, gastric cancer and other tumors 9, 10. However, very few studies have explored the prognostic value of AFR and AGR among patients with ESCC 11, 12.

The aim of the present study was to explore the correlation between preoperative AFR and AGR and clinicopathological variables and their influence on the prognosis of patients with ESCC after esophagectomy and to develop predictive nomograms containing these variables. Subsequently, the performance of the nomograms was compared with the currently used AJCC TNM staging system.

## Methods

### Patients

The medical records of consecutively admitted patients who underwent radical esophagectomy for histologically diagnosed ESCC at Tianjin Medical University Cancer Institute and Hospital between January 2005 and March 2013 were retrospectively reviewed. This study was approved by the Research Ethics Committee of Tianjin Medical University Cancer Institute and Hospital, and written informed consent was exempted due to the retrospective nature of the study.

The eligibility criteria were as follows: (1) tumors located in the thoracic esophagus, (2) histopathologically confirmed ESCC, (3) no history of another synchronous malignancy, (4) no identifiable distant metastasis, (5) no preoperative chemotherapy or radiotherapy, (6) underwent radical resection, (7) staging of tumors as pT1-4a N0-3 M0, and (8) complete medical and follow-up records were available. Patients with hematologic malignancies, chronic inflammatory disease, autoimmune disease or clinical evidence of acute and/or chronic infection and patients who received anticoagulant therapy, blood or albumin transfusions before treatment were excluded from the study.

Routine preoperative evaluation included physical examination, laboratory tests, upper gastrointestinal endoscopy with biopsy, selective endoscopic ultrasound, CT scan from the head to upper abdomen, ultrasonography of neck and abdomen, and barium swallow examination. Operative fitness was determined by clinical assessment, electrocardiography, pulmonary function testing, arterial blood gas and nutritional assessment, as clinically indicated.

All patients underwent a right-sided transthoracic esophagectomy with systematic two-field or three-field lymphadenectomy. After surgery, all patients were staged based on the 7th edition of the AJCC TNM staging system for esophageal carcinoma 13.

### Laboratory measurements of inflammatory and nutritional-based scores

Peripheral blood samples were collected before breakfast within the two weeks before the operation. The neutrophil, lymphocyte, and platelet counts were obtained by an automatic blood cell counter (XE-5000, Sysmex, Kobe, Japan). The level of preoperative fibrinogen was measured by an automatic coagulation analyzer (CS-5100, Sysmex Inc., Japan), and the albumin concentration was obtained by the liver function test.

The NLR was defined as the ratio of absolute neutrophil counts to absolute lymphocyte counts. The PLR was defined as the ratio of absolute platelet counts to absolute lymphocyte counts as previously described. The AFR was calculated as the serum albumin level divided by the plasma fibrinogen level. The AGR was calculated as the serum albumin level divided by the serum globulin level.

### Follow-up

After curative surgery, the follow-ups were first carried out within 3 months after the baseline evaluation and subsequently repeated within 6 months thereafter. The intervals were not absolute and follow-up may have been conducted early due to the patient's clinical situation or the suspicion of tumor recurrence or metastasis. All patients received a routine examination at each visit, including clinical, laboratory, and imaging examinations. The clinical attendance records were used to identify patients who had defaulted from clinic visits, and efforts were made via direct telecommunication with the patients or their family to verify their survival status. Patients lost to follow-up were censored from the analysis of survival rates. Overall survival (OS) was calculated from the date of surgery to the date of death or the last follow-up.

### Statistical analysis

For all the statistical analyses, two statistical software programs were used: SPSS version 17.0 (SPSS, Inc., Chicago, IL) and R version 4.0.2 (The R Project for Statistical Computing, Vienna, Austria; http://www.r-project.org/). X-tile software version 3.6.1 (Yale University, New Haven, CT, USA) was used to determine the optimal cutoff values of these biomarkers for predicting 5-year OS by the minimal *P*-value approach 14. The frequency of categorical variables was compared with the chi-squared test or Fisher's exact test. Survival curves were plotted and calculated via the Kaplan-Meier method with the log-rank test. Possible prognostic factors affecting survival were examined by means of univariate analysis. Multivariate analysis was performed using the Cox proportional hazards regression model incorporating significant covariates determined by univariate analysis. The hazard ratios (HRs) and 95% confidence intervals (CIs) were calculated to assess the relationship between survival time and prognostic factors. Based on the independent prognostic variables, a graphic nomogram was subsequently constructed to predict the probability of 3- and 5-year OS using the rms package from R.

Harrell's concordance index (C-index), which was calculated using the Survival package (http://cran.r-project.org/package=Survival) in R software, was used to compare the predicted accuracy of the nomogram 15. C-index intervals between 0.50 and 0.70 denoted a low accuracy, intervals between 0.71 and 0.90 denoted a medium accuracy and intervals over 0.90 denoted a high accuracy. A calibration curve was plotted using the actual and predicted incidence values to estimate the concordance between the observed OS and the predicted OS. The clinical usefulness of the nomogram models was determined using decision curve analysis (DCA) to quantify the net benefits versus the traditional AJCC staging system 16. A two-tailed* P* value <0.05 was considered statistically significant.

## Results

### Patient and tumor characteristics

The baseline clinicopathological characteristics of the 641 included ESCC patients are summarized in Table 1. Of these patients, 525 (81.9%) were men and 116 (18.1%) were women, with a median age of 61 (ranging 33~88) years. Four hundred forty (68.6%) of these patients had a history of smoking. There were 37 (5.8%), 446 (69.6%), and 158 (24.6%) patients with tumors located in the upper, middle and lower thoracic esophagus, respectively. With respect to histological type, 51 (8.0%) patients were well differentiated, 479 (74.7%) patients were moderately differentiated and 111 patients (17.3%) were poorly differentiated or undifferentiated. In accordance with the 7^th^ edition TNM staging system, 33 (5.1%) patients had pT1 disease, 119 (18.6%) tumors involved the muscularis propria (T2), 260 (40.6%) tumors involved the adventitia (T3) and 229 (35.7%) tumors involved the serosa without invasion of adjacent structures (T4a). The median number of total harvested lymph nodes was 17 (range: 4~78) per patient. Histological examination of the postsurgical specimens showed that 281 (43.8%) patients had lymph node metastasis. In addition, with regard to TNM staging, there were 35 (5.5%) patients with stage I disease, 246 (38.4%) with stage II disease, and 360 (56.2%) with stage III disease. A total of 260 (40.6%) patients underwent curative surgery alone, while 381 (59.4%) patients underwent surgery combined with postoperative adjuvant treatment.

### The optimal thresholds for these biomarkers

**Figure 1:**
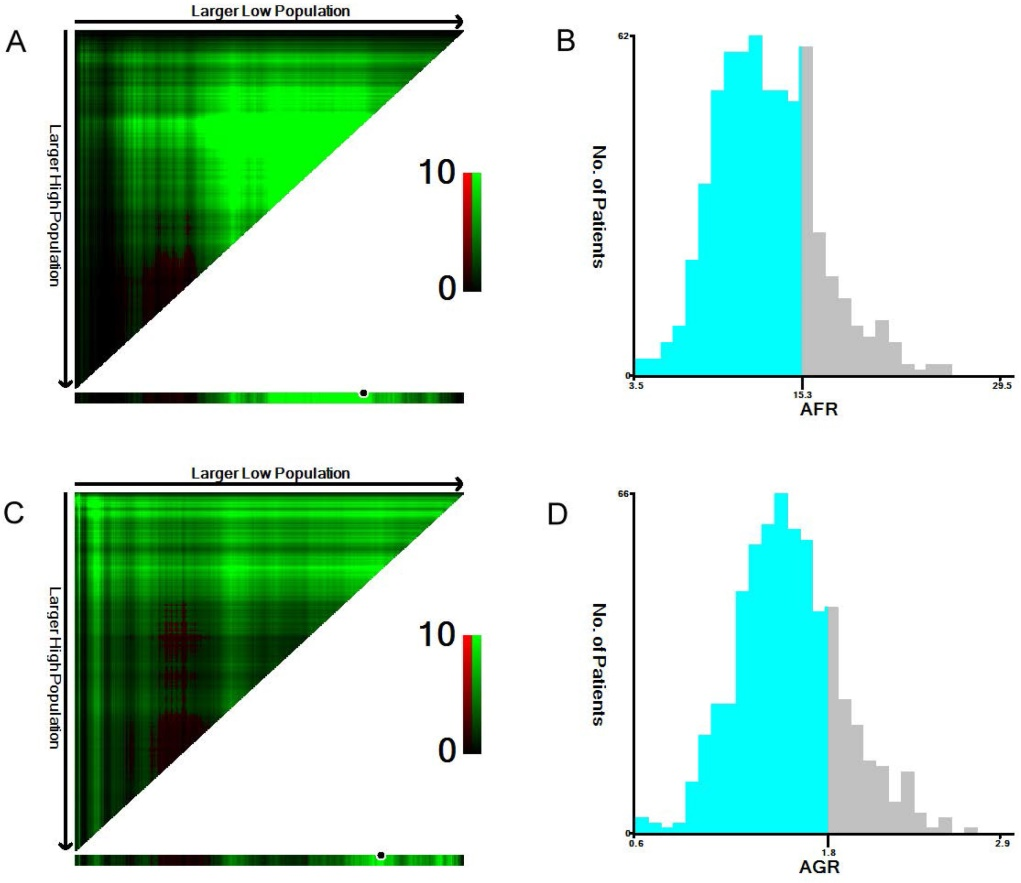
** The optimal cutoff values of preoperative AFR (A, B) and AGR (C, D) in 641 patients with ESCC using X-tile software.** The data were represented graphically in a right-triangular grid where each point represents the data from a given set of divisions (A, C). The plots showed the χ^2^ log-rank values produced, dividing them into two groups according to the cutoff point. The optimal cutoff points were determined to be 15.3 and 1.8 by locating the brightest pixel on the X-tile plot. The histogram shows the distribution of the number of patients (B, D).

The median NLR, PLR, fibrinogen, albumin, AFR and AGR values were 2.3, 129.2, 3.7, 44.3, 12.9, and 1.6, respectively. Using X-tile software, the optimal cutoff points for predicting 5-year OS were identified as 1.9 for NLR, 140.0 for PLR, 3.4 for fibrinogen, 45.6 for albumin (**Supplementary Figure 1**), 15.3 for AFR and 1.8 for AGR (**Figure 1**).

Therefore, the patients were classified into two groups according to the cutoff values as follows: a low NLR (< 1.9, n=278) group or a high NLR (≥ 1.9, n=363) group; a low PLR (<140.0, n=417) group or a high PLR (≥140.0, n=224) group; a low fibrinogen (<3.4, n=296) group or a high fibrinogen (≥3.4, n=345) group; a low albumin (<45.6, n=390) group or a high albumin (≥45.6, n=251) group; a low AFR (<15.3, n=487) group or a high AFR (≥15.3, n=154) group; and a low AGR (<1.8, n=508) group or a high AGR (≥1.8, n=133) group.

### Associations of AFR and AGR with clinicopathological variables

The associations of AFR and AGR with the clinicopathological variables of the patients are summarized in **Table 1**. Low AFR was significantly associated with tumor size, NLR, PLR, pT status, and TNM stage (all *P* <0.05). Low AGR was negatively associated with gender, age, tumor size, PLR, pT status, and TNM stage (all *P* <0.05).

### Univariate and multivariate survival analyses

Of these patients, 582 (90.8%) attended follow-up with a median follow-up period of 36.0 months (range, 3~144 months). The 1-, 3-, and 5-year overall survival rates were 82.1%, 54.0%, 41.9%, respectively. The median survival time of all patients was 43 months (95% CI: 35.57~50.43).

**Figure 2:**
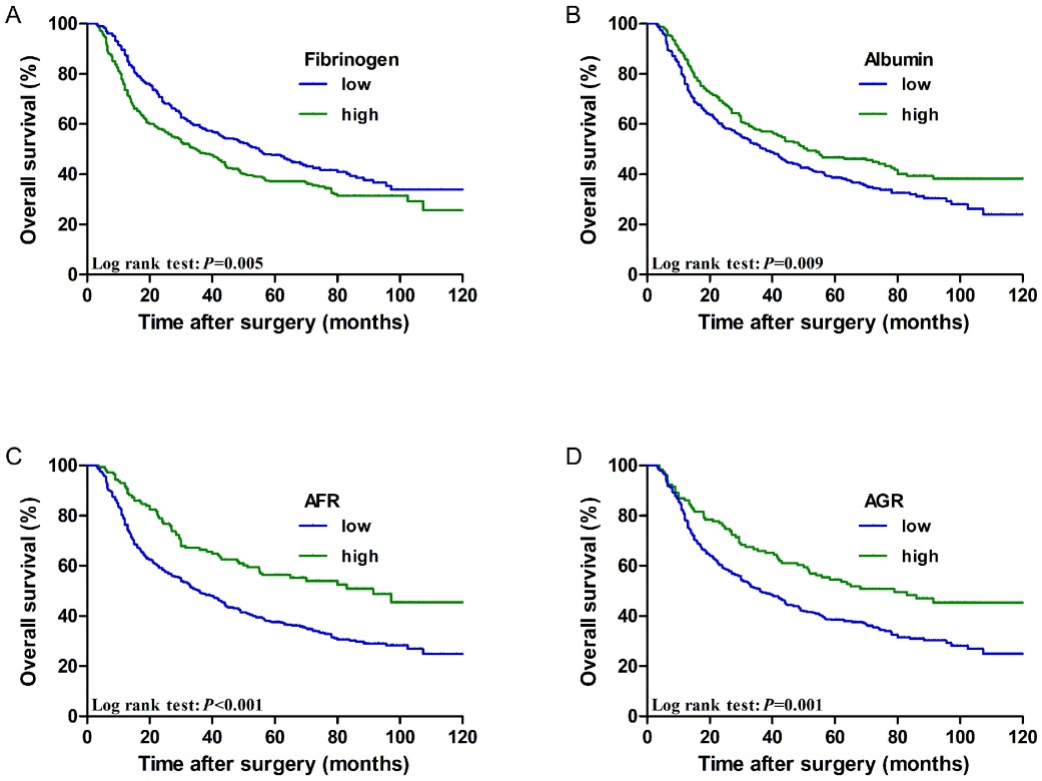
Cumulative 5-year overall survival curves for ESCC patients stratified by (**A**) fibrinogen (47.6% vs. 37.1%, *P*=0.005), (**B**) Albumin (38.7% vs. 46.7%, *P*=0.009), (**C**) AFR (56.3% vs. 37.6%, *P* < 0.001), and (**D**) AGR (54.5% vs. 38.4%, *P*=0.001).

The low fibrinogen group showed better performance regarding the 5-year OS rate of patients than the high fibrinogen group (47.6% vs. 37.1%, *P*=0.005). However, the low albumin group showed worse performance with regard to the 5-year OS rate of patients than the high albumin group (38.7% vs. 46.7%, *P*=0.009). Furthermore, the 5-year OS rate was better in patients with a high AFR than in those with a low AFR (56.3% vs. 37.6%, *P* < 0.001). The 5-year OS rate was better in patients with a high AGR than in those with a low AGR (54.5% vs. 38.4%, *P*=0.001). Survival curves of patients according to the above biomarkers are shown in** Figure 2**.

To identify the risk factors affecting postoperative OS, we evaluated the inflammatory and nutrition-based biomarkers and clinicopathologic factors in univariate and multivariate analyses. Univariate analysis showed that the factors significantly associated with OS were age (*P*=0.021), smoking history (*P*=0.041), tumor size (*P*< 0.001), pT status (*P*< 0.001), pN status (*P*< 0.001), NLR (*P*= 0.013), PLR (*P*= 0.003), fibrinogen (*P*= 0.005), albumin (*P*= 0.009), AFR (*P*< 0.001) and AGR (*P*= 0.001), whereas gender (*P*=0.163), tumor location (*P*=0.643), histological type (*P*=0.054) and postoperative adjuvant treatment (*P*=0.083) were not significantly correlated with OS. AFR was incorporated into the first step of the multivariate analysis, and was identified as an independent risk factor (*P*= 0.018). AGR was added to the model in the second step of the multivariate analysis and was found to be insignificant (*P*= 0.154). When both AFR and AGR were included in the third step of the multivariate analysis, only AFR (HR: 0.690, 95% CI = 0.495~0.960, *P* = 0.028) was confirmed to be significant with other variables.

### Subgroup analysis

The inflammatory and nutritional-based scores were related to TNM stage. Therefore, we further stratified the patients into two groups based on tumor stage. The survival curves for patients with stage I-II disease are shown in**Supplementary Figure 2**, and the 5-year OS rates were similar between the low and high fibrinogen groups (61.5% vs. 50.7%, *P*=0.114) and the low and high albumin groups (54.6% vs. 58.3%, *P*=0.260). The 5-year OS rate was markedly worse in patients with the low AFR group than in those with the high AFR group (50.6% vs. 69.5%, *P* = 0.002). Likewise, patients in the low AGR group had a markedly worse 5-year OS rate than those in the high AGR group (51.3% vs. 69.5%, *P* = 0.004).

The survival curves for patients with stage III disease are shown in **Supplementary Figure 3**, and the 5-year OS rate was remarkably worse in the low AFR group than in the high AFR group (28.6% vs. 38.1%, *P*=0.036). However, the 5-year OS rates among the low and high fibrinogen, albumin, and AGR groups did not differ significantly (all *P* > 0.05).

### Prognostic nomogram for OS

**Figure 3:**
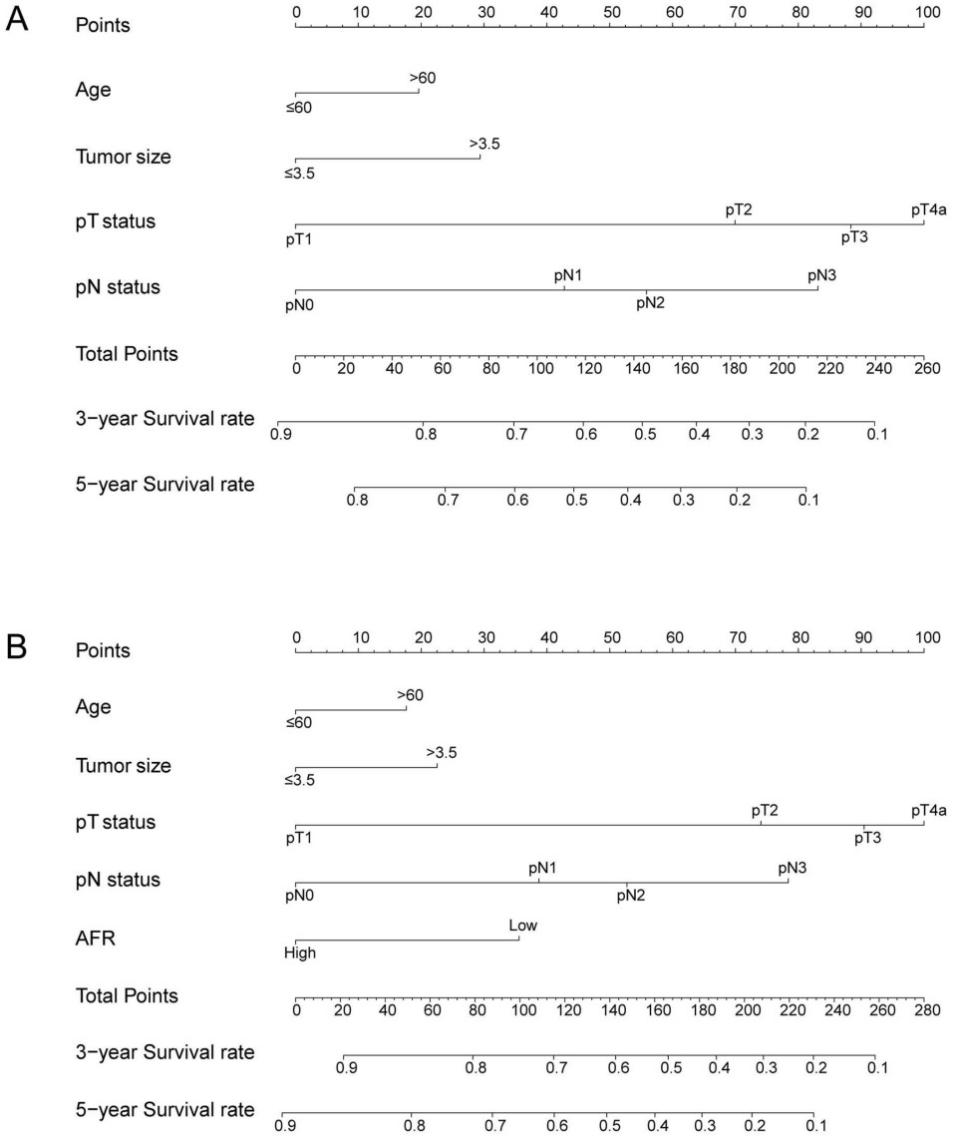
The nomogram for predicting 3-year and 5-year OS in ESCC patients who underwent surgical resection without AFR (A, C-index = 0.656) and with AFR (B, C-index = 0.677).

**Figure 4:**
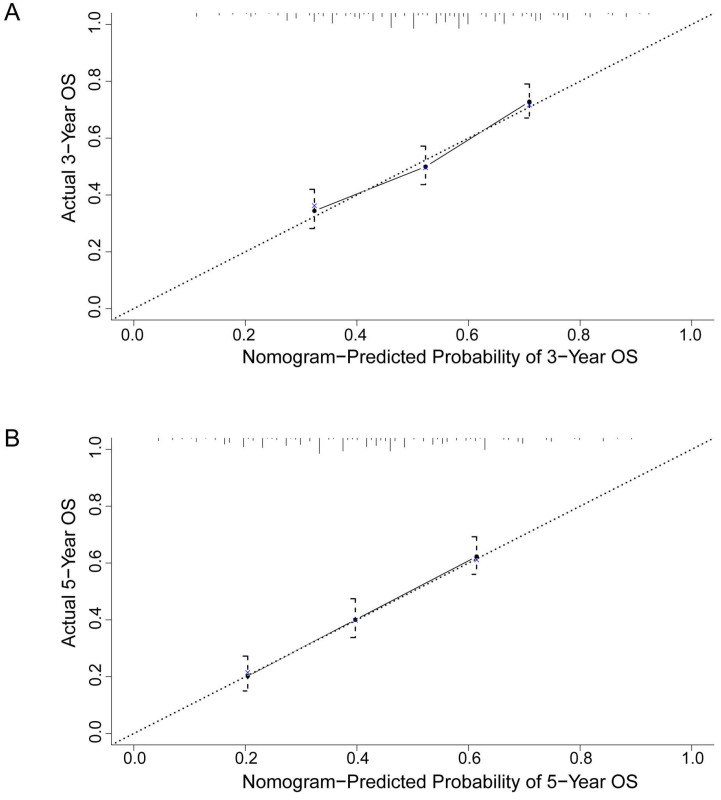
The calibration curves for predicting 3-year (**A**) and 5-year (**B**) overall survival of ESCC patients after radical esophagectomy. The nomogram-predicted probability of overall survival is plotted on the X-axis, and the actual overall survival is plotted on the Y-axis. The dotted line represents the ideal correlation between predicted and actual survival.

To better predict the prognosis of ESCC patients after radical resection, prognostic nomograms for pivotal clinicopathological variables were established to predict 3- and 5-year OS, and the predicted accuracy of the model was evaluated by Harrell's C-index. The prognostic nomogram that integrated all significant independent factors from multivariate analysis is shown in** Figure 3**. The C-index of the prognostic nomogram with AFR was 0.677, which represented higher accuracy than that without AFR (C-index: 0.656) in predicting 5-year OS after initial surgery. Moreover, the calibration plot showed optimal agreement between the prediction by the nomogram and the actual observed probability of survival at 3- or 5- years (**Figure 4**).

### Decision curve analysis

**Figure 5:**
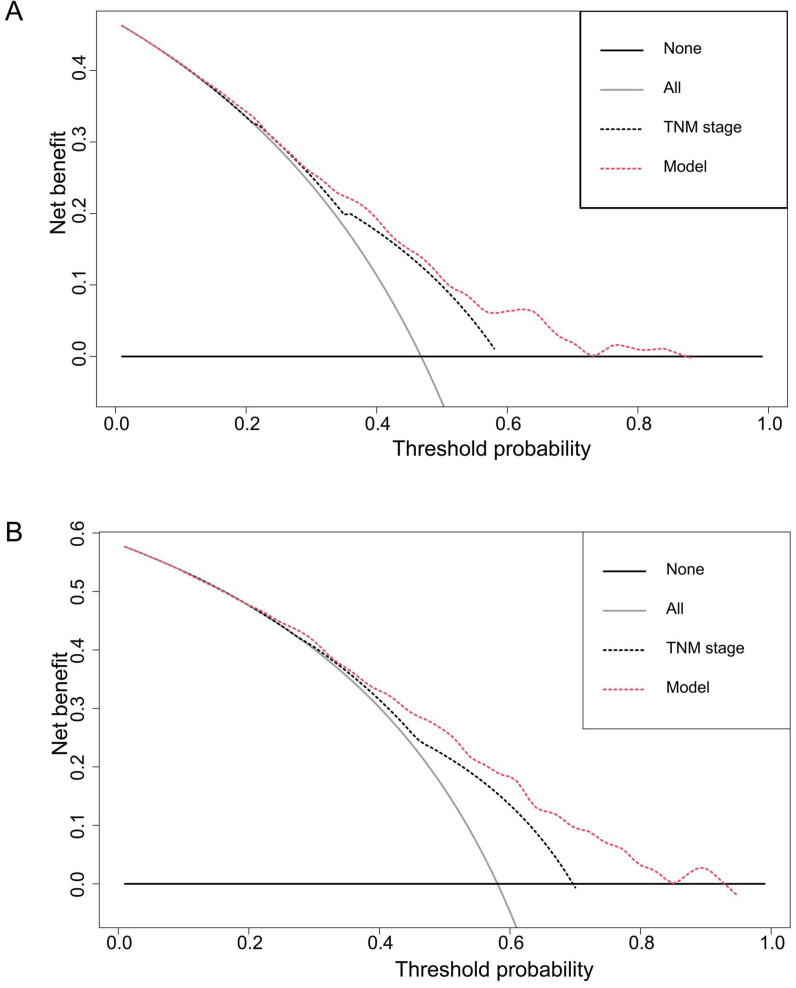
** Decision curve analyses of the nomogram and the AJCC TNM staging system for 3-year (A) and 5-year (B) overall survival prediction.** The nomograms showed higher net benefit to the AJCC staging system with a wide range of threshold probabilities. The horizontal coordinates represent the threshold probability, and the vertical coordinates represent the net benefit rate. The red dashed line represents the DCA of the nomogram, and the black dashed line represents the DCA of the 7th TNM staging system. The assumption that all of the patients will die is shown with a grey solid line, and the assumption that all of the patients will survive is indicated with a black solid line.

DCA, a novel method to evaluate prediction models from the perspective of clinical consequences, revealed that the established nomogram achieved greater net clinical benefits than the 7^th^ AJCC TNM staging system (**Figure 5**). The constructed nomogram has good clinical applicability in predicting the OS of patients with ESCC.

## Discussion

Esophageal cancer is the sixth leading cause of cancer-related deaths worldwide 17. In recent years, the prognosis of patients with esophageal cancer has remained poor despite improvements in surgical techniques, rational lymphadenectomies, and aggressive therapeutic strategies. It is widely recognized that the staging system for the accurate classification of malignant tumors must be feasible, precise, and reproducible for prognostic stratification without stage migration 18. The results of the current study show that AFR is an independent prognostic factor, which is better than other inflammation-related indicators in predicting the outcomes of ESCC patients.

Investigators have demonstrated that neutrophils, lymphocytes, plasma fibrinogen and serum albumin play prominent roles in cancer-related inflammation and are considered to be associated with patient outcomes 19. Recently, the roles of some integrated inflammation- and nutrition-based indicators in the prognosis assessment of patients with malignancies have been reported, including NLR/Alb, CRP/Alb, PNI, etc. 20, 21. Thus, we speculated that AFR or AGR, which reflect inflammation and nutrition status, would be novel indicators of the prognosis of malignant tumors.

Zhang et al. 9 conducted a retrospective study involving 545 patients with NSCLC and found that a low level of AGR was an independent risk factor for mortality. Postoperative adjuvant chemotherapy can significantly improve the prognosis of patients with AGR ≤ 1.43. Du et al. 22 proposed that a low level of AGR can predict the low survival rate of patients with undifferentiated nasopharyngeal carcinoma. A retrospective study analyzed 365 elderly patients with gastric cancer and pointed out that the preoperative AFR level is a useful factor in predicting postoperative complications after radical laparoscopic gastrectomy 23. Chen et al. 24 also pointed out that AFR before resection can be an independent prognostic factor for NSCLC patients, and a higher AFR can increase OS and DFS. However, the clinical burden and prognostic impact of these two indicators in ESCC remain to be clarified.

Recently, a few studies on AFR and AGR in esophageal cancer have been published. Matsuda S et al. 11 showed that the survival rate of esophageal cancer patients with lower AFR scores was significantly reduced. Studies have also shown that a reduction in preoperative AGR levels is associated with the depth of tumor invasion, positive lymph node metastasis and poor prognosis in esophageal cancer patients 12, 25. Although the relationships between the above two indicators and tumor invasion, metastasis and patient survival have been reported, it is not yet clear which indicator is superior for the prognostic evaluation of ESCC.

In our present study, the clinical and prognostic value of AFR and AGR in 641 patients with ESCC were analyzed. Our results showed that low levels of AFR and AGR are closely related to a number of aggressive tumor phenotypes, including larger tumor size, deeper tumor invasion, and advanced TNM staging. Moreover, low AFR and AGR were shown to be predictive of worse outcomes, which is consistent with previously published reports 11, 12, 25. Multivariate analysis identified preoperative AFR, not AGR, as an independent prognostic variable, suggesting that AFR was superior to AGR in predicting the prognosis of ESCC patients. NLR and PLR have been proven to play important roles in the prognostic evaluation of a variety of malignancies 26. In this study, NLR and PLR were not identified as independent predictors that affect the prognosis of ESCC patients, regardless of whether AFR or AGR were included in the multivariate analysis at the same time or separately. To a certain extent, this result shows that AFR plays a greater role in the prognostic evaluation of ESCC patients than other inflammation-related indicators.

TNM staging is currently recognized as an important factor in evaluating the prognosis of patients with ESCC 27. We further explored the prognostic impact of these variables in different pathological stage subgroups in the current investigation. The AFR maintained its prognostic value in OS prediction for all the subgroups of ESCC patients, indicating the reliable prognostic value of the AFR.

In addition, we created visual nomograms based on clinical variables and AFR, which could improve the prediction accuracy of individual prognosis. The C-index of the nomogram including AFR was higher than that without AFR, indicating that AFR could improve the predictive efficacy of the nomogram for the prognosis of ESCC. DCA plots are a method to directly compare benefits and harms on the same scale 16. The curves of the DCA in our study showed that the established nomograms have more net benefit than the AJCC staging system in predicting OS among ESCC patients.

The possible mechanisms underlying the correlation of AFR or AGR with tumor occurrence and development are as follows. First, albumin is the main protective component against carcinogenic factors produced by nitrosamines and aflatoxins. It can specifically inhibit the growth of cancer cells by stabilizing DNA replication, strengthening natural immunity and regulating hormone homeostasis 28. Notably, the secretion of albumin can be inhibited by some proinflammatory cytokines, including IL-1, IL-6 and tumor necrosis factor (TNF)-α 29. Second, globulin is produced by immune organs and reflects the immune and inflammatory status of the body. It contains a large number of acute-phase proteins, such as α1-antitrypsin, α2-macroglobulin and haptoglobin, which may lead to tumor progression by damaging the immune system and changing the tumor microenvironment 30. It has been proven that the excessive production of inflammatory cytokines such as IL-6 can increase the level of fibrinogen, which can mediate cellular interactions and influence tumor cell activities, including proliferation, migration, and apoptosis 31. Moreover, fibrinogen may produce signals to upregulate the expression of growth factors, such as fibroblast growth factor-2 (FGF-2), vascular endothelial growth factor (VEGF) and platelet-derived growth factor (PDGF), thus promoting malignant tumor cell proliferation and angiogenesis 32. Fibrinogen-platelet microthrombi formed by fibrin, cancer cells, and platelets adhering to tumor cells could protect circulating malignant cells from mechanical damage and natural immunity and promote adhesion to the metastatic site to promote tumor progression and metastasis 33. An interesting animal model of Lewis lung cancer was subcutaneously inoculated in mice lacking fibrinogen, and the results showed that the number of metastatic lymph nodes and lung metastases were significantly reduced 34. A recent study reported that high levels of fibrinogen can promote tumor invasion by inducing epithelial-mesenchymal transition in ESCC cells and increasing the expression levels of p-PTEN, p-AKT, and p-mTOR 35.

To the best of our knowledge, this is the first study to simultaneously investigate the prognostic role of AFR and AGR in ESCC patients. However, there are several limitations that should be addressed. First, this is a retrospective-design, single-center study with a relatively small number of samples, which may lead to potentially inaccurate medical record data. Second, as the cutoff points of AFR and AGR vary among different studies, whether the optimal cutoff values calculated via X-tile analysis can be applied to other studies remains to be demonstrated using other independent external population-based data. Third, although the DCA plot confirms that our nomogram has more net benefit than the AJCC staging system, it is still a purely statistical method and cannot replace prospective clinical trials. Therefore, these results must be validated independently in multicenter and prospective studies with a large cohort of patients.

In summary, our study revealed that preoperative AFR is a novel and promising inflammation-based indicator that could independently predict patient outcomes with better performance than other inflammation‑based variables and guide the formulation of clinical treatment strategies. In addition, a reliable prognostic nomogram based on clinical parameters and AFR can improve its predictive value in patient OS.

## Supplementary Material

Supplementary figures.

## Figures and Tables

**Table 1:** The associations of clinicopathologic categories with AFR and AGR in patients with ESCC

Categories	Cases	AFR		*P*	AGR		*P*
		Low	High		Low	High	
**Gender**				0.321			**0.011**
Male	525	403	122		406	119	
Female	116	84	32		102	14	
**Age (years)**				0.467			**< 0.001**
≤ 60	300	224	76		217	83	
> 60	341	263	78		291	50	
**Smoking history**				0.181			0.106
None	201	146	55		167	34	
Yes	440	341	99		341	99	
**Tumor location**				0.194			0.813
Upper	37	26	11		28	9	
Middle	446	333	113		353	93	
Lower	158	128	30		127	31	
**Tumor size (cm)**				**< 0.001**			**0.005**
≤ 3.5	292	191	101		217	75	
> 3.5	349	296	53		291	58	
**Histological grade**				0.269			0.253
G1	51	35	16		36	15	
G2	479	371	108		385	94	
G3	111	81	30		87	24	
**NLR**				**< 0.001**			0.150
< 1.9	278	184	94		213	65	
≥ 1.9	363	303	60		295	68	
**PLR**				**< 0.001**			**0.006**
<140.0	417	297	120		317	100	
≥140.0	224	190	34		191	33	
**pT status**				**0.003**			**< 0.001**
T1	33	24	9		22	11	
T2	119	80	39		77	42	
T3	260	191	69		206	54	
T4	229	192	37		203	26	
**pN status**				0.264			0.095
N0	360	264	96		273	87	
N1	177	140	37		146	31	
N2	64	53	11		54	10	
N3	40	30	10		35	5	
**TNM stage**				**0.001**			**0.001**
I-II	281	195	86		206	75	
III	360	292	68		302	58	

AFR, albumin/fibrinogen ratio; AGR, albumin/globulin ratio; ESCC, esophageal squamous cell carcinoma; G1, well differentiated; G2, moderately differentiated; G3, poorly differentiated/undifferentiated; NLR, neutrophil/lymphocyte ratio; PLR, platelet/lymphocyte ratio; TNM, tumor node metastasis.

**Table 2:** Univariate and multivariate Cox proportional hazards regression models for overall survival in patients with ESCC

Categories	Univariate analysis		Multivariate analysis 1		Multivariate analysis 2		Multivariate analysis 3	
	HR (95% CI)	*P* value	HR (95% CI)	*P* value	HR (95% CI)	*P* value	HR (95% CI)	*P* value
Gender	0.826 (0.631~1.081)	0.163						
Age	1.270 (1.037~1.556)	**0.021**	1.263 (1.027~1.551)	**0.027**	1.243 (1.011~1.529)	**0.039**	1.249 (1.015~1.536)	**0.036**
Smoking history	1.261 (1.009~1.574)	**0.041**	1.178 (0.940~1.476)	0.156	1.213 (0.966~1.524)	0.096	1.199 (0.955~1.506)	0.118
Tumor location	0.957 (0.793~1.154)	0.643						
Tumor size	1.569 (1.277~1.927)	**< 0.001**	1.269 (1.021~1.579)	**0.032**	1.291 (1.039~1.605)	**0.021**	1.268 (1.019~1.576)	**0.033**
Histological type	1.217 (0.997~1.487)	0.054						
pT status	1.455 (1.284~1.649)	**< 0.001**	1.284 (1.135~1.453)	**< 0.001**	1.273 (1.124~1.441)	**< 0.001**	1.275 (1.126~1.443)	**< 0.001**
pN status	1.502 (1.353~1.667)	**< 0.001**	1.427 (1.282~1.589)	**< 0.001**	1.427 (1.282~1.589)	**< 0.001**	1.423 (1.278~1.584)	**< 0.001**
NLR	1.299 (1.058~1.596)	**0.013**	1.124 (0.889~1.419)	0.329	1.139 (0.901~1.440)	0.275	1.127 (0.892~1.424)	0.318
PLR	1.375 (1.118~1.692)	**0.003**	1.203 (0.953~1.518)	0.120	1.207 (0.956~1.524)	0.114	1.190 (0.942~1.503)	0.145
Fibrinogen	1.336 (1.090~1.637)	**0.005**	0.948 (0.741~1.213)	0.670	1.085 (0.874~1.349)	0.459	0.935 (0.731~1.198)	0.597
Albumin	0.758 (0.615~0.935)	**0.009**	0.921 (0.739~1.146)	0.460	0.891 (0.717~1.107)	0.296	0.943 (0.755~1.178)	0.606
AFR	0.557 (0.427~0.726)	**< 0.001**	0.673 (0.485~0.934)	**0.018**	---	---	0.690 (0.495~0.960)	**0.028**
AGR	0.628 (0.481~0.819)	**0.001**	---	---	0.813 (0.611~1.081)	0.154	0.848 (0.636~1.130)	0.259
Adjuvant treatment	0.829 (0.671~1.025)	0.083						

ESCC, esophageal squamous cell carcinoma; HR, hazard ratio; CI, confidence interval; NLR, neutrophil/lymphocyte ratio; PLR, platelet/lymphocyte ratio; AFR, albumin/fibrinogen ratio; AGR, albumin/globulin ratio.

## Abbreviations

ESCC: esophageal squamous cell carcinoma
AFR: albumin/fibrinogen ratio
AGR: albumin/globulin ratio
NLR: neutrophil/lymphocyte ratio
PLR: platelet/lymphocyte ratio
C-index: concordance index
DCA: decision curve analysis
NSCLC: non-small cell lung cancer
OS: Overall survival
HR: hazard ratio
CI: confidence interval
FGF-2: fibroblast growth factor-2
VEGF: vascular endothelial growth factor
PDGF: platelet-derived growth factor
TNM: tumor node metastasis
G1: well differentiated
G2: moderately differentiated
G3: poorly differentiated/undifferentiated
