# Supplementary material for: Prognostic Significance of the Preoperative Albumin/Fibrinogen Ratio in Patients with Esophageal Squamous Cell Carcinoma after Surgical Resection
Source: J Cancer. 2021 Jun 16;12(16):5025–34. doi: 10.7150/jca.58022 (PMC8247378; doi:10.7150/jca.58022)
Supplement: Supplementary file 1 — Supplementary figures. [file jcav12p5025s1.pdf]

Prognostic Significance of the Preoperative Albumin/Fibrinogen Ratio in Patients with Esophageal Squamous Cell Carcinoma after Surgical Resection

Hongdian Zhang[1], Peng Ren[1], Mingquan Ma[1], Xiaolei Zhu[1], Kai Zhu[1], Wanyi Xiao[1], Lei Gong[1], Peng Tang[1], Zhentao Yu[1, 2]

Supplementary Figures

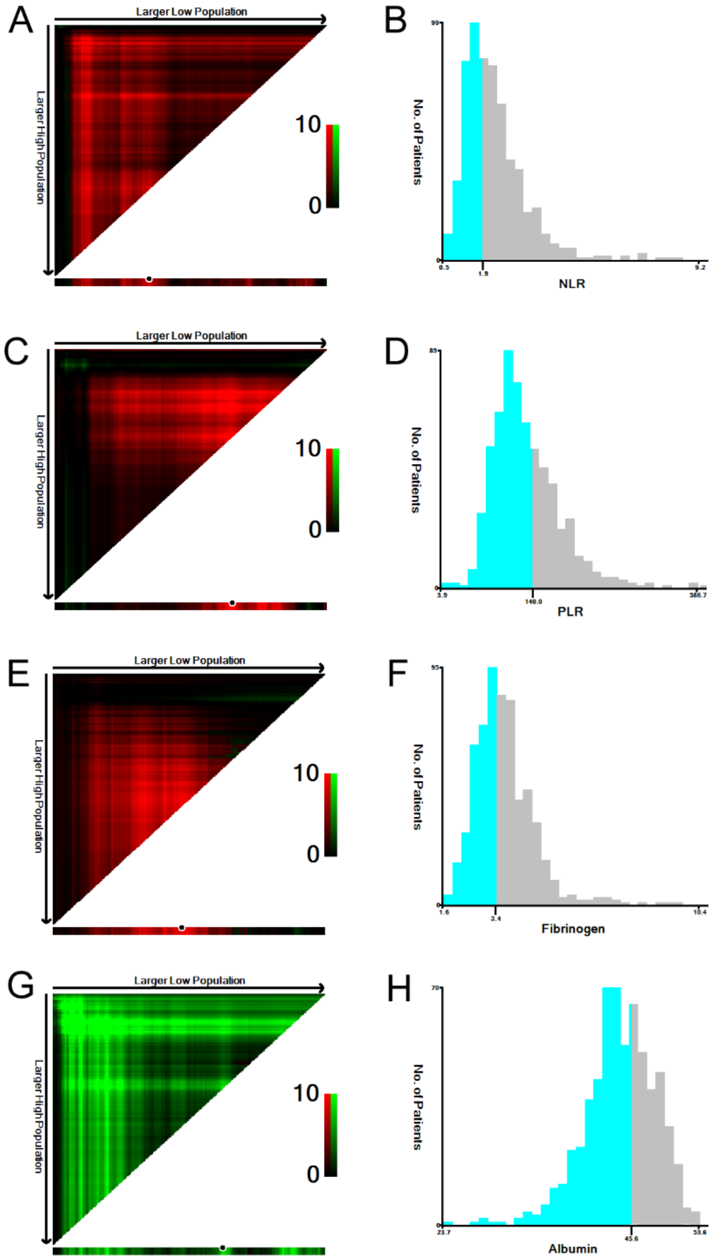

Supplementary Figure 1. The optimal cutoff values of preoperative NLR (A-B), PLR (C-D), fibrinogen (E-F), and albumin (G-H) in 641 patients with ESCC using X-tile software.

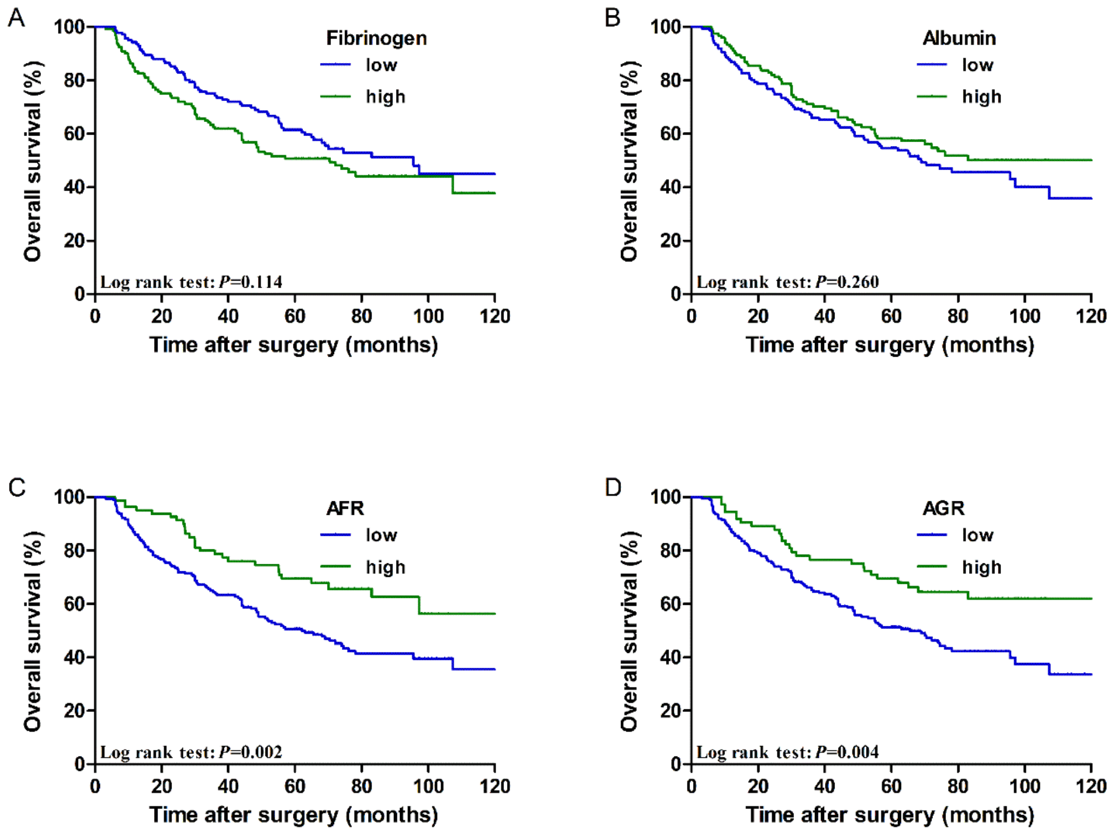

Supplementary Figure 2. Cumulative 5-year overall survival curves for stage I-II ESCC patients according to (**A**) fibrinogen (61.5% vs. 50.7%, *P*=0.114), (**B**) albumin (54.6% vs. 58.3%, *P*=0.260), (**C**) AFR (50.6% vs. 69.5%, *P* = 0.002), and (**D**) AGR (51.3% vs. 69.5%, *P* = 0.004).

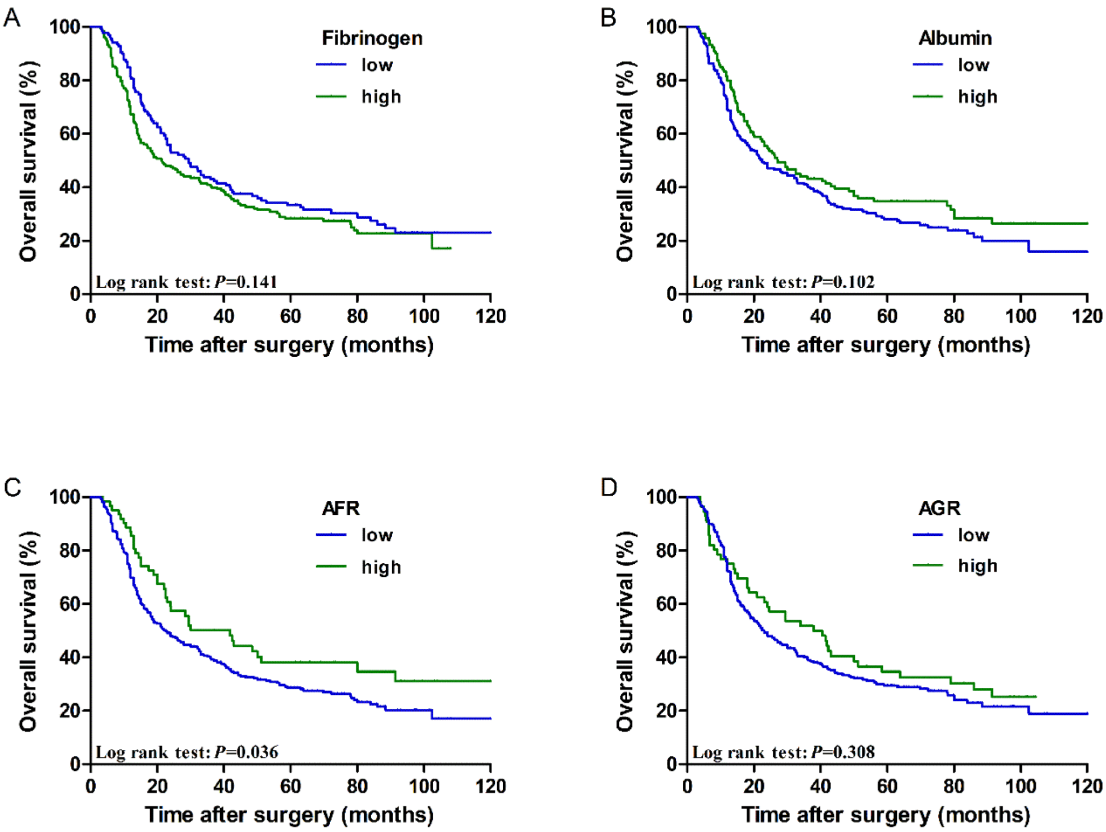

Supplementary Figure 3. Cumulative 5-year overall survival curves for stage III ESCC patients according to (**A**) fibrinogen (33.3% vs. 28.2%, *P* = 0.141), (**B**) albumin (28.0% vs. 34.7%, *P* = 0.102), (**C**) AFR (28.6% vs. 38.1%, *P* = 0.036), and (**D**) AGR (29.5% vs. 34.6%, *P* = 0.308).
